# RECLAIM—A retrospective, multicenter observational study aimed at enabling the development of artificial intelligence-driven prognostic models for disease progression in multiple sclerosis

**DOI:** 10.3389/fneur.2025.1557947

**Published:** 2025-05-16

**Authors:** Jelle Praet, Lina Anderhalten, Giancarlo Comi, Dana Horakova, Tjalf Ziemssen, Patrick Vermersch, Carsten Lukas, Koen Van Leemput, Marjan Steppe, Noemí Manero, Ella Kadas, Alexis Bernard, Jean van Rampelbergh, Erik de Boer, Vera Zingler, Dirk Smeets, Annemie Ribbens, Friedemann Paul

**Affiliations:** ^1^icometrix NV, Leuven, Belgium; ^2^Experimental and Clinical Research Center (ECRC), A Cooperation Between the Max Delbrück Center for Molecular Medicine in the Helmholtz Association and Charité - Universitätsmedizin Berlin, Berlin, Germany; ^3^Department of Neurorehabilitative Sciences, Milan, Italy; ^4^Department of Neurology, Vita Salute San Raffaele University, Milan, Italy; ^5^Department of Neurology and Center of Clinical Neuroscience, First Faculty of Medicine, Charles University and General University Hospital in Prague, Prague, Czechia; ^6^Center of Clinical Neuroscience, Department of Neurology, University Clinic Carl Gustav Carus, TU Dresden, Dresden, Germany; ^7^Univ. Lille, InsermU1172 LilNCog, CHU Lille, FHU Precise, Lille, France; ^8^Institute of Neuroradiology, St. Josef Hospital, Ruhr-University Bochum, Bochum, Germany; ^9^Athinoula A. Martinos Center, Department of Radiology, Massachusetts General Hospital, Charlestown, MA, United States; ^10^Department of Neuroscience and Biomedical Engineering, Aalto University, Espoo, Finland; ^11^Department of Computer Science, Aalto University, Espoo, Finland; ^12^European Charcot Foundation, Brussels, Belgium; ^13^SYNAPSE Research Management Partners, Madrid, Spain; ^14^Nocturne GmbH, Berlin, Germany; ^15^AB Science, Clinical Development, Paris, France; ^16^Imcyse SA, Liège, Belgium; ^17^Bristol-Myers Squibb Company Corp., Princeton, NJ, United States; ^18^F. Hoffmann-La Roche Ltd., Product Development Medical Affairs, Neuroscience, Basel, Switzerland; ^19^Experimental and Clinical Research Center (ECRC), Charité - Universitätsmedizin Berlin, Corporate Member of Freie Universität Berlin and Humboldt-Universität zu Berlin, Berlin, Germany; ^20^Max Delbrück Center for Molecular Medicine in the Helmholtz Association (MDC), Berlin, Germany; ^21^Neuroscience Clinical Research Center (NCRC), Charité - Universitätsmedizin Berlin, Corporate Member of Freie Universität Berlin and Humboldt-Universität zu Berlin, Berlin, Germany; ^22^Department of Neurology with Experimental Neurology, Charité - Universitätsmedizin Berlin, Corporate Member of Freie Universität Berlin and Humboldt-Universität zu Berlin, Berlin, Germany

**Keywords:** data, AI model, disease worsening, biomarker, observational study, real-world data, clinical trial, multiple sclerosis

## Abstract

Multiple sclerosis (MS) is characterized by a progressive worsening of disability over time. As many regulatory-cleared disease-modifying treatments aiming to slow down this progression are now available, a clear need has arisen for a personalized and data-driven approach to treatment optimization in order to more efficiently slow down disease progression and eventually, progressive disability worsening. This strongly depends on the availability of biomarkers that can detect and differentiate between the different forms of disease worsening, and on predictive models to estimate the disease trajectory for each patient under certain treatment conditions. To this end, we here describe a multicenter, retrospective, observational study, aimed at setting up a harmonized database to allow the development, training, optimization, and validation of such novel biomarkers and AI-based decision models. Additionally, the data will be used to develop the tools required to better monitor this progression and to generate further insights on disease worsening and progression, patient prognosis, treatment decisions and responses, and patient profiles of patients with MS.

## Introduction

### Background

Multiple sclerosis (MS) is a devastating immune-mediated disorder of the central nervous system (CNS) resulting in neurological disability and a wide range of physical and cognitive impairments (1). Irreversible worsening of clinical disability can occur at any stage of the disease and is driven mainly by two processes. First, patients with MS (pwMS) may accumulate disability due to acute inflammatory relapses, known as Relapse Associated Worsening (RAW), and second, pwMS may accumulate disability not associated with relapses which is referred to as Progression Independent of Relapse Activity (PIRA) (2).

While there are currently about 20 regulatory-approved Disease-Modifying Treatments (DMTs) available, most of them were developed to target acute inflammatory relapses, thus lesion formation. Therefore, and not surprisingly, clinical decision-making and treatment optimization are highly focused on these acute inflammatory lesions. Additionally, all existing guidelines on the use of DMTs in MS are based on expert judgment and differ across countries (3, 4). As of 2017, the first DMT for the treatment of progressive MS was regulatory approved in the US and Europe (5), and nowadays more clinical trials aiming to treat progressive MS are on their way. The latter is driven by accumulating evidence that PIRA can drive disability accumulation already at early disease stages (6) and even in the absence of RAW (7). Even though PIRA entered the spotlights only recently and is therefore not fully understood yet, brain and spinal cord atrophy, as well as chronic active lesions (i.e., paramagnetic rim lesions), have been shown to substantially contribute to PIRA (8, 9).

The heterogeneity of MS combined with the availability of multiple DMTs (with limited scope) indicate the need for a data-driven and personalized approach to treatment optimization, to more efficiently slow down disease progression and disability worsening. While early diagnosis and patient-level prognostic modeling are important to provide data-driven personalized recommendations on treatment optimization, the ability to disentangle and monitor the disability accumulation due to RAW or PIRA will be key to achieving the best possible long-term outcomes (10, 11). The latter strongly depends on the availability of biomarkers, including imaging markers of different entities, that can detect and differentiate between different forms of disability worsening and underlying disease aspects, enabling the early detection of PIRA throughout the disease course.

### Study objectives

The study described in this paper is being conducted within the scope of the EU-funded research project “Clinical impact through AI-assisted MS Care” (CLAIMS^1^). CLAIMS aims to address the urgent need for more data-driven and personalized care for pwMS. To this end, CLAIMS will develop, validate, and seek regulatory approval for a companion platform that provides a holistic view of each patient by visualizing both existing and new biomarker data, as well as predict disease trajectories under different treatment scenarios.

The objective of the RECLAIM study is to collect and harmonize a substantial retrospective dataset on pwMS for research purposes. This dataset will be used for the development, training, optimisation and validation of novel biomarkers and AI-based decision models that support prognosis of individual pwMS’ disease course and treatment responses in a real-world setting. Additionally, the data will be used to develop new tools to better monitor this progression and to generate further insights on disease worsening and progression, patient prognosis, treatment decisions and responses, and patient profiles of pwMS.

## Methods and analysis

### Study design

RECLAIM is a multicenter, retrospective, observational study, aimed at setting up a harmonized database to allow the secondary use of data for research and development purposes. This database will include real-world healthcare data from routine clinical practice, structured data from observational studies as well as control arm data from clinical trials.

### Participants

We expect the inclusion of the majority of records of ±7,000 patients from real-world routine clinical care and observational studies (6 clinical centers in 4 countries). This will be supplemented with data from randomized controlled trials from ±4,000 patients (coming from 4 pharmaceutical companies). In addition to the focus on data from pwMS, the foreseen retrospective dataset will also include real-world clinical data from patients with Clinically Isolated Syndrome (CIS) and Radiologically Isolated Syndrome (RIS) as well as Neuromyelitis Optica Spectrum Disorder (NMOSD) and Myelin oligodendrocyte glycoprotein antibody-associated disease (MOGAD). The inclusion of CIS and RIS patients allows us to gain insights into and develop models that consider the very early stages of MS. Despite the recommendations on standardized brain and spinal cord imaging in MS diagnosis and monitoring (12), misdiagnosis of MS in the presence of mimicking CNS pathologies can occur. Given that both NMOSD and MOGAD have overlapping disease patterns and MRI presentations when compared with MS, the inclusion of data from NMOSD and MOGAD patients is relevant. By incorporating this data, we will be able to build more robust prognostic models for MS specifically, as we can identify the differences in clinical and radiographic presentation and disability worsening that set NMOSD and MOGAD apart from MS.

### Inclusion/exclusion criteria

Inclusion criteria that will be applied:

- Participants must have a confirmed diagnosis of MS, NMOSD, MOGAD, CIS or RIS.
- Participant (or participant’s legal representative) has previously signed and dated an informed consent form (ICF) for the secondary use of their data, or assent form. Alternatively, the secondary use of the patient’s data is allowed following Institutional Review Board (IRB)/Ethical Committee (EC) approval in accordance with national and local subject privacy regulations.

Participants will be excluded according to the following exclusion criteria:

- Participants under 18 years of age will be excluded.
- Other unspecified reasons that, in the opinion of the Investigator or Joint Steering Committee, make the participant unsuitable for study participation.

### Endpoints

The accomplishment of a sufficiently large, harmonized database for research purposes will be determined by:

- The number of patients from each institution who have contributed data to the database.
- The number of patients from each institution whose data was mapped to the common data model of the harmonized database.
- The number of patients from the control arms of clinical trials who have contributed data to the database.
- The data completeness of each variable in the harmonized database.

The accomplishment of a sufficiently high quality, harmonized database for research purposes will be determined by:

- The representativeness of the harmonized dataset for the MS patient population as evaluated by age range, gender balance, the distribution of country of residence, the distribution of race/ethnicity and the distribution of educational level.
- The validity of the data through an assessment of the amount of erroneous or impossible data entries for each variable.
- The temporal uniformity of each institution’s data over time as assessed by the number of changes to variables over time (addition of new variables or variables no longer being captured, alterations to how variables are captured).
- The temporal uniformity of the harmonized dataset over time as assessed by the average time between subsequent assessments of each variable.
- The presence of contextual information on standard data gathering and analysis processes of each institution.
- The presence of a unique and pseudonymized patient ID for all data of each patient, allowing to link such data of each patient.

The feasibility of using the harmonized database for the development of the anticipated novel biomarkers and AI-based decision models, will be determined by:

- The temporal uniformity of MRI data over time as assessed by the comparability of MRI scans and the average time between subsequent MRI assessments for each patient.
- The percentage of MRI data sets that are compliant with the MAGNIMS-CMSC-NAIMS acquisition guidelines.
- The percentage of MRI data sets for which the automated quality control process of icobrain ms did not indicate any quality issues upon analysis.
- The percentage of patients with a complete disease-modifying treatment history available, from the date of diagnosis to the current day.
- The percentage of patients with a complete disease history available, from the date of diagnosis to the current day.
- The validity and temporal uniformity for disability assessment as clinically determined by EDSS, Functional systems score, T25FWT, 9HPT and SDMT. Each of these scores will be assessed individually for the amount of erroneous or impossible data entries, as well as for the average time between subsequent assessments of each variable.

### Outcome parameters

While there is no minimum nor a limit of the observational time required for patients, on average, retrospective data spanning up to 10 years is anticipated, provided the data quality is sufficiently high. When choosing data and variables of interest for the study, relevance for the development and optimisation of the AI-based models was balanced with feasibility of acquiring this data during routine clinical care. The categories of data we collect consist of:

- Patient-specific data: demographics, comorbidities and risk factors
- Disease-specific data: disease history, disease status, relapse history and visual tests
- Subclinical data: magnetic resonance imaging (MRI) from brain and spinal cord, Optical coherence tomography (OCT) and Evoked potentials (EP)
- Treatment history: disease-modifying treatments (DMT) and non-pharmaceutical treatments (NPT)

### Sample size

Given the objective of the study, no specific sample size calculations can be performed. Typical AI algorithms need at least data of 300 subjects for training of simple tasks. Phase III RCT trials in MS recruit on average 500–1,500 patients to evaluate statistical differences between different treatment strategies. In this project, we envision novel insights while evaluating the influence of specific confounding factors and treatment strategies (with 19 DMTs available in the clinical market in the EU). Moreover, not all DMT’s will be evenly represented: older drugs will inevitably be overrepresented and newer drugs underrepresented; therefore, the data sample needs to be large enough to have sufficient data also on the latter treatments. Additionally, the fact that real-world clinical data are often of suboptimal quality, with many irregularities, further complicates things. As such, we believe the anticipated number of participants to be justified and required.

### Data flow

**Figure 1:**
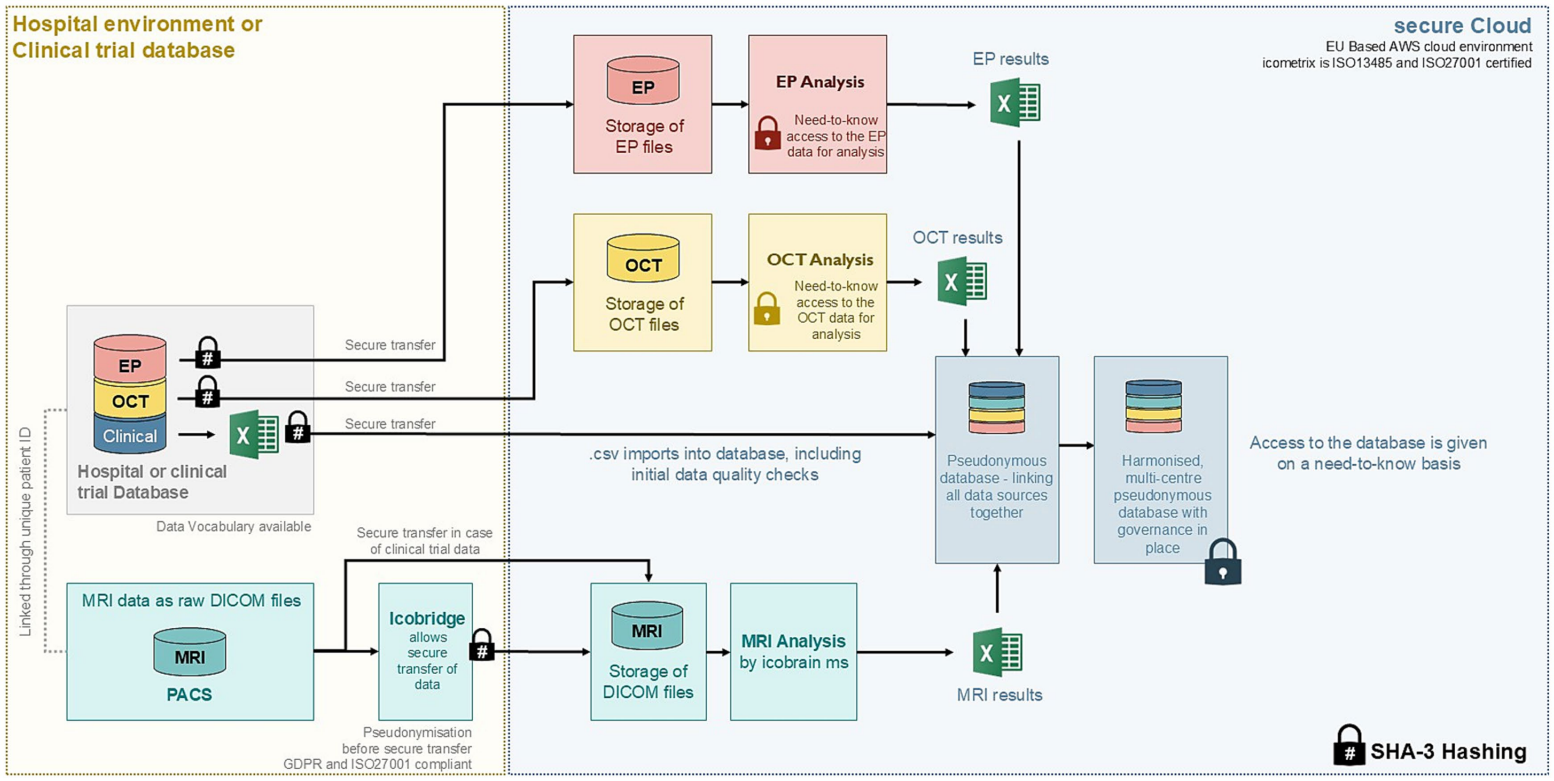
High-level overview of the RECLAIM study data flow. MRI, Magnetic Resonance Imaging; OCT, Optical Coherence Tomography; EP, Evoked Potential; PACS, Picture Archiving and Communication System; DICOM, Digital Imaging and Communications in Medicine; AWS, Amazon Web Services; SHA-3, Secure Hash Algorithm 3; GDPR, General Data Protection Regulation.

The data collection, storage and handling will be coordinated by the study sponsor compliant with ISO 27001 (information security management), 21 CFR Part 11, HIPAA, and GDPR. The flow of data is shown in Figure 1. While clinical trial data is *a priori* fully anonymized, all data from hospital environments will be pseudonymised prior to leaving the hospital. Data transfer will be done through secure data transfer tools using the industry-standard TLS (Transport Layer Security) encryption protocol. For MRI data in particular, the icobridge software will be used (for more information on icobridge, we refer to https://icobridge.icometrix.com/).

As a general approach, we are using a SHA-3 hashed version of the “Patient Identifier” (PID-number) provided by the hospital to correctly link all of a patient’s pseudonymised data together, including the MRI, OCT and EP data.

The clinical routine data will be captured in a pre-formatted character-separated values (.csv) spreadsheet and, as far as possible, exported directly from the electronic database or the Electronic Medical Record system in which the data is stored at the clinical center. No data is expected from paper records that have not been previously digitalized.

MRI data as raw DICOM files will be as much as possible retrieved by the icobridge software directly from the hospital’s Picture Archiving and Communication System (PACS) or a desktop application. In this process, the images are automatically pseudonymized and PID numbers will be automatically SHA-3 hashed. Raw OCT data will be similarly pseudonymised before data transfer and analysis. Different formats of raw OCT data are accepted, such as, but not limited to. E2E, .vol, .img, DICOM and others, depending on the OCT device vendor. Raw EP data will likewise be pseudonymised and then securely transferred to the cloud environment for analysis.

### Data analysis

Quantitative assessments will be performed on the subclinical imaging data and these will be added to the database alongside the raw data. The MRI data will be analysed using the icobrain ms software,^2^ a CE-marked and 510 k FDA-cleared AI-driven software solution providing insights into brain volume loss and MS lesion load (13, 14). Similarly, OCT source data will be analysed by Nocturne GmbH^3^ and Neuroquantic srls. For EP data, we will store all data together with normative data of the source laboratory. Then, all waveforms will be reconstructed and measured centrally by Neuroquantic srls. Additionally, during the next research steps within or beyond the project, additional characteristics and/or imaging features can be extracted from these imaging data to gain novel insights in the biomarkers that drive disease worsening.

### Data harmonization

All data collected, including the centrally analyzed data, will undergo the necessary data curation to guarantee a database that complies with the FAIR principles (findable, accessible, interoperable and reusable). In this context, all data will be mapped to a common data structure, originally based on the MS Data Alliance (MSDA) Core Dataset (15), but expanded for the purposes of this study. A data dictionary will be made available for the common data structure used.

### Biomarker and AI model development

The data collected in this study will be used in pseudonymised form for research aligned with the CLAIMS project goals, which includes developing novel biomarkers for the assessment and differentiation of MS disease worsening as well as for the development of predictive AI models. While additional research questions and statistical analyses can be included upon agreement of the joint Steering Committee, the initial analysis will focus on:

- Assessment and development of AI-driven analysis pipelines for (imaging) biomarkers characteristic for disease worsening related to RAW and PIRA (e.g., brain atrophy, slowly evolving lesions, spinal cord lesions, relation with patient-reported outcomes, etc.)
- Development and validation of an AI-driven biomarker-based MS progression model, the development and validation of an imaging-focused generative model to predict brain characteristic evolution, and the development and validation of an interventional model for treatment optimization.

### Study report

A descriptive characterization of the patient population in terms of demographics, MS history, MRI, treatment history, and more will be published after completion of the harmonized database. All results, insights and developments generated through the use of this database will also be made publicly available and disseminated widely through open-access publications where possible.

### Ethics statement

This study will be performed in alignment with the ethical principles outlined in the Declaration of Helsinki. The study will be conducted in full conformance with ICH-GCP and the laws and regulations of the countries in which the research is conducted. An independent ethics advisor is appointed to provide support and guidance on all ethical, privacy and security issues. If required by local IRB, EC, or other oversight body, prior to any data collection and data sharing under this protocol, written informed consent or assent with the center-specific approved Informed Consent File will be obtained from the patient or patient’s legally authorized representative, as applicable, in accordance with local practice and regulations. Each participating investigator will obtain IRB or EC approval of the protocol or notice of exemption prior to starting the project.

## Discussion

Real-world clinical data has the advantage that it often allows to study larger groups of patients over long timeframes (versus clinical trial data), but one of the major difficulties in using real-world clinical data is that it is known to often be incomplete (e.g., variables are not captured each visit), to contain errors, to contain duplicates, etc. In addition, this data is captured during routine clinical care, therefore large variations in the time between subsequent assessments are to be expected. Also, each contributing clinical partner will have differences in how they perform routine clinical care for pwMS, thus the data available will also differ between the individual centers. The endpoints chosen for this study reflect the quality assessments that will be performed on these data, assessing its usefulness for the development of AI models and biomarkers related to disease progression.

Data representativeness will be assessed as one of the endpoints as we are working with clinical centers in Europe, which might result in an underrepresentation of certain demographic groups of patients. This could potentially lead to bias in the AI-driven models, resulting in suboptimal AI model predictions for these groups of patients. Secondly, in an observational setting, disease-modifying therapies are given to patients according to guideline recommendations and patient presentation. Observational data is therefore biased by these guidelines, and appropriate measures will be put in place to control for this bias when developing the AI models.

Typically, clinical trial data is of higher quality, in terms of data points available and the quality of the data set, which is tremendously beneficial when developing an AI model. For example, MRI scans that are acquired in clinical trials use a harmonized and up-to-date protocol and includes all necessary sequences. In addition, follow-up scans are obtained within a specific timeframe, on the same scanner, and with the same sequences. However, as ultimately the goal is to develop predictive models that can work in a clinical routine setting, it is important to also include real-world data during the development and validation of these AI models. Otherwise, when built based on a clinical trial data set only, these models will not be able to be incorporated into routine clinical care.

A major focus of the RECLAIM study is the collection of MRI data, which is generally available for most patients and therefore forms a core element of any AI model to be developed. By conducting an AI-driven centralized analysis of the raw MRI data, we significantly reduce variability due to different analysis methods. The AI tools used to analyse this MRI data is able to efficiently handle the different MRI scanners and acquisition protocols that were used to acquire the MRI. In addition, software-based analysis of MRI is much faster, less prone to errors, and much more sensitive to subtle changes when compared to manual reading (16). A similar approach will be used for the analysis of the OCT and EP data, which are affected by similar issues.

## Conclusion

The RECLAIM study will set up a harmonized database consisting of real-world clinical care data supplemented with control arm clinical trial data. This dataset will be used for the development, training, optimization, and validation of novel biomarkers and AI-based decision models that support the prognosis of individual MS patients’ disease courses and treatment responses in a real-world setting. Additionally, the data will be used to develop tools to monitor disease progression and to generate further insights on disability worsening, patient prognosis, treatment decisions and responses, and patient profiles of pwMS.

## Footnotes

^1^ www.claims.ms/

^2^ www.icometrix.com

^3^ http://nocturne.one/
